# An MRI-Based Radiomics Model for Predicting the Benignity and Malignancy of BI-RADS 4 Breast Lesions

**DOI:** 10.3389/fonc.2021.733260

**Published:** 2022-01-28

**Authors:** Renzhi Zhang, Wei Wei, Rang Li, Jing Li, Zhuhuang Zhou, Menghang Ma, Rui Zhao, Xinming Zhao

**Affiliations:** ^1^ Department of Diagnostic Radiology, National Cancer Center/National Clinical Research Center for Cancer/Cancer Hospital, Chinese Academy of Medical Sciences and Peking Union Medical College, Beijing, China; ^2^ School of Electronics and Information, Xi’an Polytechnic University, Xi’an, China; ^3^ College of Engineering, Boston University, Boston, MA, United States; ^4^ Faculty of Environment and Life, Beijing University of Technology, Beijing, China

**Keywords:** LASSO, BI-RADS 4, breast lesion, magnetic resonance imaging, radiomics

## Abstract

**Objectives:**

The probability of Breast Imaging Reporting and Data Systems (BI-RADS) 4 lesions being malignant is 2%–95%, which shows the difficulty to make a diagnosis. Radiomics models based on magnetic resonance imaging (MRI) can replace clinicopathological diagnosis with high performance. In the present study, we developed and tested a radiomics model based on MRI images that can predict the malignancy of BI-RADS 4 breast lesions.

**Methods:**

We retrospective enrolled a total of 216 BI-RADS 4 patients MRI and clinical information. We extracted 3,474 radiomics features from dynamic contrast-enhanced (DCE), T_2_-weighted images (T_2_WI), and diffusion-weighted imaging (DWI) MRI images. Least absolute shrinkage and selection operator (LASSO) and logistic regression were used to select features and build radiomics models based on different sequence combinations. We built eight radiomics models which were based on DCE, DWI, T_2_WI, DCE+DWI, DCE+T_2_WI, DWI+T_2_WI, and DCE+DWI+T_2_WI and a clinical predictive model built based on the visual assessment of radiologists. A nomogram was constructed with the best radiomics signature combined with patient characteristics. The calibration curves for the radiomics signature and nomogram were conducted, combined with the Hosmer-Lemeshow test.

**Results:**

Pearson’s correlation was used to eliminate 3,329 irrelevant features, and then LASSO and logistic regression were used to screen the remaining feature coefficients for each model we built. Finally, 12 related features were obtained in the model which had the best performance. These 12 features were used to build a radiomics model in combination with the actual clinical diagnosis of benign or malignant lesion labels we have obtained. The best model built by 12 features from the 3 sequences has an AUC value of 0.939 (95% CI, 0.884-0.994) and an accuracy of 0.931 in the testing cohort. The sensitivity, specificity, precision and Matthews correlation coefficient (MCC) of testing cohort are 0.932, 0.923, 0.982, and 0.791, respectively. The nomogram has also been verified to have calibration curves with good overlap.

**Conclusions:**

Radiomics is beneficial in the malignancy prediction of BI-RADS 4 breast lesions. The radiomics predictive model built by the combination of DCE, DWI, and T_2_WI sequences has great application potential.

## Introduction

The 2020 Global Cancer Report released by the International Agency for Research on Cancer (IARC) shows that female breast cancer has replaced lung cancer as the most common cancer in the world with an increase of approximately 2.3 million new cases (11.7%) throughout the year (1). Breast cancer is also estimated to top the list of new morbidity and mortality among all types of cancers in women. During treatment, the diagnosis of benign or malignant breast lesions has become the most basic and important step in the treatment of breast diseases.

According to the breast cancer screening guidelines of the National Comprehensive Cancer Network (NCCN) and BI-RADS, suspicious lesions can be classified into 6 categories (2). The fourth category of breast disease is defined as a type of breast lesions with suspicious malignancy and uncertain pathological types. The probability of being malignant is 2%–95%, although this type of breast disease has a further classification of 4a, 4b, and 4c, because of the large range of the possibility of the existence of malignant lesion, all patients with BI-RADS 4 breast diseases are recommended to undergo biopsy of suspicious areas to clarify their pathological properties (3). Because of the blurry qualitative characteristics of BI-RADS 4 breast diseases, we can see that most patients with BI-RADS 4 of breast diseases are overdiagnosed and treated with the puncture case analysis, which requires a certain degree of trauma to the body. Moreover, the clinical diagnosis inevitably has a certain false-positive rate and missed diagnosis rate (4, 5). Therefore, we propose a hypothesis to establish a predictive radiomics model based on the patients’ preoperative imaging information, thereby avoiding patients with benign breast lesions from undergoing invasive pathological testing.

Computer-aided diagnosis methods based on medical imaging have been increasing in clinical application value in recent years. This method only needs to mark the abnormal signs, and then perform common image processing on this basis to get the diagnosis result. Therefore, the concept of radiomics came into being (6). Radiomics is a research method that extracts high-throughput image features from medical images and conducts quantitative research. DCE, DWI, and T_2_WI are three routine methods for the diagnosis and observation of breast diseases. Breast DCE imaging has high sensitivity in breast cancer screening for women who have accumulated breast cancer risk for more than 20%–25% (7). However, its specificity depends on a variety of external factors, such as the professional skills of the reader or the method of using quantitative techniques. DWI can characterize the three-dimensional fluidity of water in the body and indirectly detect and visualize the microstructure (8). DWI and apparent diffusion coefficient (ADC) have been successfully applied to the clinical diagnosis and screening of breast cancer. Compared with the average specificity of 80% in DCE for breast cancer diagnosis, the average specificity of the combined diagnosis of DCE and DWI can reach 89.2% (9). Therefore, radiologists mainly use the combination of these two technologies in the diagnosis of breast cancer. T_2_WI is usually used to exclude cysts, intramammary lymph nodes, and other benign breast lesions (10). One of the most advantageous characteristics of T_2_WI is that signal strength is directly related to the underlying disease state for most breast cancer lesions mainly showing uneven or slightly high signal on T_2_WI MRI, while the surrounding tissues show low or medium signal (11). As existing research shows that the T_2_WI MRI image has a strong ability to interpret the pathology and diagnosis of breast diseases (12). Research combining T_2_WI MRI, DCE MRI, and DWI MRI to increase the actual diagnosis efficiency also picture a wider application of combining multiple MRI features in the field of breast diagnosis (13, 14).

It can be seen from the above results that the combined application of different MRI images can play a greater practical role in the diagnosis of breast diseases. This study aims to establish an auxiliary diagnosis prediction model that can be used to predict benign and malignant breast lesions by combining MRI images with three sequences of DCE, DWI, and T_2_WI.

## Materials and Methods

This study had been reviewed and approved by the Ethics Committee of Cancer Hospital, Chinese Academy of Medical Sciences, and had been in line with the Declaration of Helsinki. All patients participating in this study waived the requirement of informed consent. The image processing methods had met the terms and conditions mentioned in the Transparent Reporting of a Multivariable Model for Individual Prognosis or Diagnosis (TRIPOD), Image Biomarker Standardization Initiative (IBSI), Checklist for Artificial Intelligence in Medical Imaging (CLAIM).

### Patients

The patient information for this research study comes from the picture archiving and communication system (PACS) of the Cancer Hospital of the Chinese Academy of Medical Sciences. Due to the following criteria, we continuously enrolled a total of 216 research subjects from 230 patients who received diagnosis at our hospital from September 2018 to December 2019. Each of the patients was classified with the fifth version of BI-RADS guidelines. The criteria for inclusion in the group were as follows: (i) Patients have been diagnosed with a BI-RADS type IV breast lesion. (ii) The pathological diagnoses results were confirmed by puncture pathology diagnoses. (iii) Patients have complete MRI images in PACS with axial DCE, DWI, and T_2_WI sequences obtained before patients underwent biopsy.

All the in-group patients were separated into a training cohort of 144 patients and a testing cohort of 72 patients divided by scan time from early to late according to the ratio of 2:1. We use leave-one-out cross-validation (LOOCV), and the training cohort is used to train and validate the model. Testing cohort is used to test the performance of the model.

### Semiautomatic Image Segmentation and Feature Extraction

We collected image information of the 216 enrolled patients, which were composed of three kinds of MRI images: DCE, DWI, and T_2_WI. All patients’ MRI scans were completed before they underwent biopsy. Patients in training and testing cohorts were scanned using the same equipment. The DCE sequences were obtained by a higher axial resolution T1-weighted DCE imaging with a temporal resolution of 90 s. The DWI sequences were obtained by a DWI sequence with two *b*‐values (0–1,000 s/mm^2^). The T_2_WI sequences were obtained from a higher axial resolution T_2_WI turbo spin-echo sequence. All scans were done at a magnetic field strength of 3.0 T.

**Figure 1:**
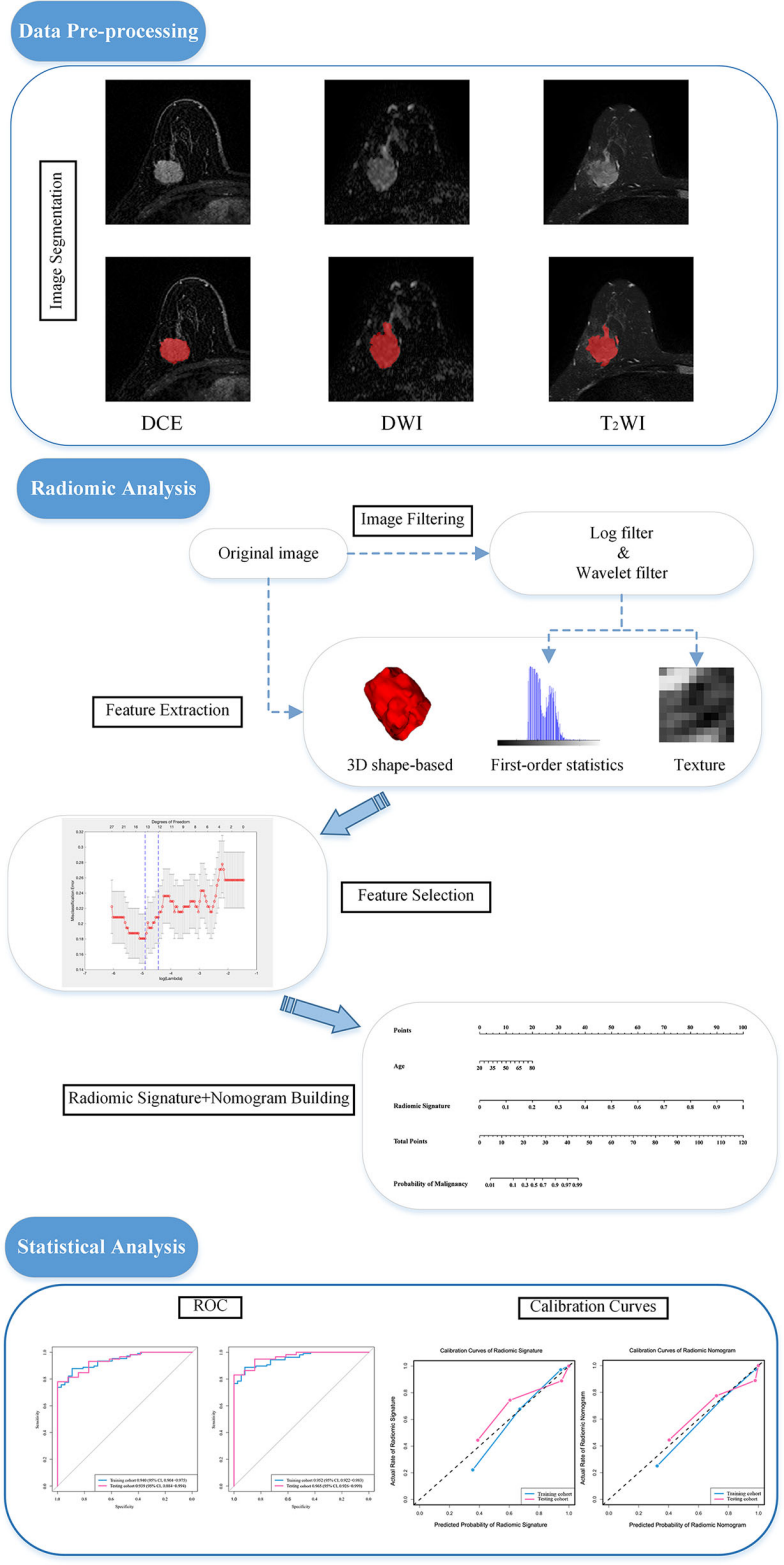
Image segmentation and the procedure of developing a predictive model. The experiment is divided into three main parts: image pre-processing, image radiomic analysis and statistical analysis. Radiomic analysis includes image feature extraction and feature filtering. DCE, dynamic contrast enhanced imaging; DWI, diffusion weighted imaging; T_2_WI, _2_ weighted imaging; ROC, receiver operating characteristic curve.

These MRI images were reviewed by 2 radiologists who were not aware of the real pathological diagnoses. They calibrated and refined the segmentation results from Radiomics (www.radiomics.net.cn) of the breast lesion regions of interest (ROI) of the patients’ pretreatment images in the DCE, DWI, and T_2_WI views. This software is a computerized semiautomatic image segmentation software with high accuracy trained by using a deep learning model. It uses the most recognized nnU-Net model framework, which is a generalized U-net framework and can obtain better training results through a careful preprocessing process and diverse network training schemes. An example of Radiomics software combined with manual fine-tuning for semiautomatic image segmentation is shown in **Figure 1**.

The specific operations of feature extraction were all done through Python (3.7.9), where the “pyradiomics” package (pyradiomics.readthedocs.io) was used in conjunction. The hyperparameters of feature extractor set as follows: Laplacian of Gaussian filter: sigma: [2.0, 3.0, 4.0, 5.0]; normalize: True; normalize scale: 100; resampled pixel spacing (1, 1, 1):; interpolator: sitkBSpline; binWidth: 5; and voxelArrayShift: 300. The other settings are default. A total of 3,474 quantitative original image features including first-order statistics features (18 features), 3D shape-based features (14 features), gray-level cooccurrence matrix features (24 features), gray-level run length matrix features (16 features), gray-level size zone matrix features (16 features), and gray-level dependence matrix features (14 features) were extracted from the corresponding regions of interest in the original images in the DCE, DWI, and T_2_WI views (15). The original images were later added with wavelet filtering and Laplace of Gaussian (LoG) filtering respectively to extract the above features. Wavelet filtering was aimed to yield 8 decompositions per level of the original images. LoG filtering was used as an edge enhancement filter which emphasized areas of gray level changes, where sigma defines how the emphasized textures are supposed to be (16, 17).

### Radiomics Signature Building

Due to the same coarse-to-fine strategy, we filtered the features to prevent the model from overfitting (18–20). First, a mono-factor analysis was performed on all features, and all features were ranked in order from smallest to largest *p*-value, and the top 5% of features were filtered out. Second, the LASSO algorithm made the image features most relevant to the BI-RADS 4 breast lesions to be filtered out by compressing the correlation coefficients of some of the features and zeroing out another part of the coefficients. Finally, the LOOCV was performed to select a model. After the above steps are completed, the coefficients of most radiomics features were compressed to zero. Then, a radiomics signature was established based on a linearly weighted combination of features with nonzero coefficients (21). The above content is the entire process of our training model. The feature extraction and selection process were implemented in Python (3.7.9).

The ability of the model to recognize benign and malignant lesions is evaluated by the drawn ROC. ROC can directly show the sensitivity (true-positive rate) and false-positive rate of the predicted results of the model. Meanwhile, the AUC value and the accuracy value are two main indicators that can be indirectly obtained from the curve. In addition, we also calculated precision and MCC metrics to evaluate the model.

### Development and Performance of the Models Built With Sequences Combinations

Based on the above modeling steps, we performed prediction model building using different combinations of the three sequences, respectively. First, we modeled the three sequences individually based on the eigenvalue data extracted from each sequence. Second, the feature data of each two sequences were combined and modeled based on this data. Finally, we modeled the data using the combined data of the three sequences. These seven different models were used to compare performance in the same computational way.

### Development and Performance of the Radiomics Nomogram

To verify that radiomics signature combined with clinical factor has a greater predictive ability, we built the radiomics nomogram (21). Through the progress of organizing the patients’ information and univariate logistic regression analysis, we selected age as the clinical factor used for radiomics nomogram building. We constructed a calibration curve for the nomogram to demonstrate its predictive efficacy.

### Imaging Diagnosis by the Radiologists

In order to further verify the application value of the prediction model, we invited two radiologists to participate in our model verification step. Both radiologists come from the Cancer Hospital of the Chinese Academy of Medical Sciences and have more than 10 years of clinical diagnosis experience. The experiment requires the two experts to combine DCE, DWI, and T_2_WI MRI images of each patient without contacting the patient and completely ignorant of the clinical information and actual diagnosis results to jointly make their pathological diagnosis of each case of whether benign or malignant breast lesion judgments. During the actual experiment, two radiologists conducted pathological identification of all patients through visual observation of images. By comparing the prediction results of our model with the diagnosis results of these two experts, we can evaluate whether this model has value in clinical application.

After obtaining the diagnosis results of the two radiologists, we used the same calculation method to calculate the accuracy, sensitivity, specificity, precision, and MCC of the radiologist’s diagnoses and compare them with the corresponding indicators of the radiomics model.

### Data and Statistical Analysis

To further evaluate the results, we plotted nomograms from the clinical information and plotted calibration curves combined with the Hosmer-Lemeshow test for the nomograms and the ROC curves of the radiomics model with the strongest performance. The p-value was calculated by a two samples *t*-test to evaluate the degree of group differentiation of the data. The above statistical evaluation work was done through R version 4.0.5 (R Foundation for Statistical Computing, Vienna, Austria).

## Results

### Clinical Characteristics of the Patients

The clinical characteristics of the patients in the training cohort and the testing cohort are shown in **Table 1**. The benign rate of breast lesion in the training cohort is 25.69% and that of the testing cohort is 18.05%. The differences in imaging devices, age, and other clinical characteristics were not statistically significant between the two cohorts.

**Table 1:** Basic clinical information of enrolled patients.

Characteristics	Training (N = 144)	Testing (N = 72)	Total (N = 216)	*P*-value
Age at surgery (years), median (range)	45 (22-72)	45 (23-78)	45 (22-78)	0.052^a^
Benign (%)	37 (25.69)	13 (18.06)	50 (23.15)	0.867^b^
Adenomatosis	9 (6.25)	3 (4.17)	12 (5.56)	
Phyllodes tumor	28 (19.44)	10 (13.89)	38 (17.59)	
Malignant (%)	107 (74.31)	59 (81.94)	166 (76.85)	0.066^b^
Invasive ductal carcinoma	67 (46.53)	28 (38.89)	95 (43.98)	
Colloid carcinoma	20 (13.89)	11 (15.28)	31 (14.35)	
Medullary carcinoma	18 (12.60)	19 (25.39)	37 (17.13)	
Neuroendocrine carcinoma	1 (0.69)	1 (1.39)	2 (0.93)	
Solid papillary carcinoma	1 (0.69)	0 (0.00)	1 (0.46)	

The differentiation in the characteristics (age when diagnosed, benignity and malignancy, pathological diagnosis) in the training cohort and the Testing cohort were evaluated. P-value less than 0.05 proves that the groups are significantly different. The above P-values show the training and the Testing cohorts are non-significantly different.

a P-value was calculated by two sample t-test.

b P-values were calculated by Fisher exact test.

### Feature Selection, Radiomics Signature Development, and Validation

Twelve features were derived from DCE, DWI, and T_2_WI MRI images with the principle of coarse-to-fine. The selected features and corresponding coefficients are listed in **Table 2**.

**Table 2:** Results of the feature selection for the model based on DCE, DWI and T_2_WI.

Sequences	Features	Coefficients
T_2_WI	Wavelet LHH glrlm long run low gray level emphasis	-0.19765
	Original glszm Gray Level Non-Uniformity Normalized	-0.52642
	Wavelet LLL glszm Small Area Low Gray Level Emphasis	-1.53857
	Wavelet LHL glszm Low Gray Level Zone Emphasis	-1.34546
	Wavelet LLH glszm Small Area Low Gray Level Emphasis	-6.49947
	Log sigma 4-0-mm-3D glrlm Long Run Low Gray Level Emphasis	-0.00238
	Log sigma 4-0-mm-3D glrlm Long Run Emphasis	-0.00004
	Wavelet LHH glszm Gray Level Non-Uniformity Normalized	-1.11352
DCE	Original glszm Small Area Emphasis	4.57208
	Wavelet LHH glcm Correlation	12.60474
	Wavelet LLL glcm Inverse Variance	-4.61787
DWI	Wavelet HHH glrlm Long Run High Gray Level Emphasis	-0.02102

The features were selected by LASSO algorithm. These coefficients show the magnitude of the weight of their corresponding characteristics in the regression model.

### Development, Performance, and Evaluation of the Prediction Models

**Figure 2:**
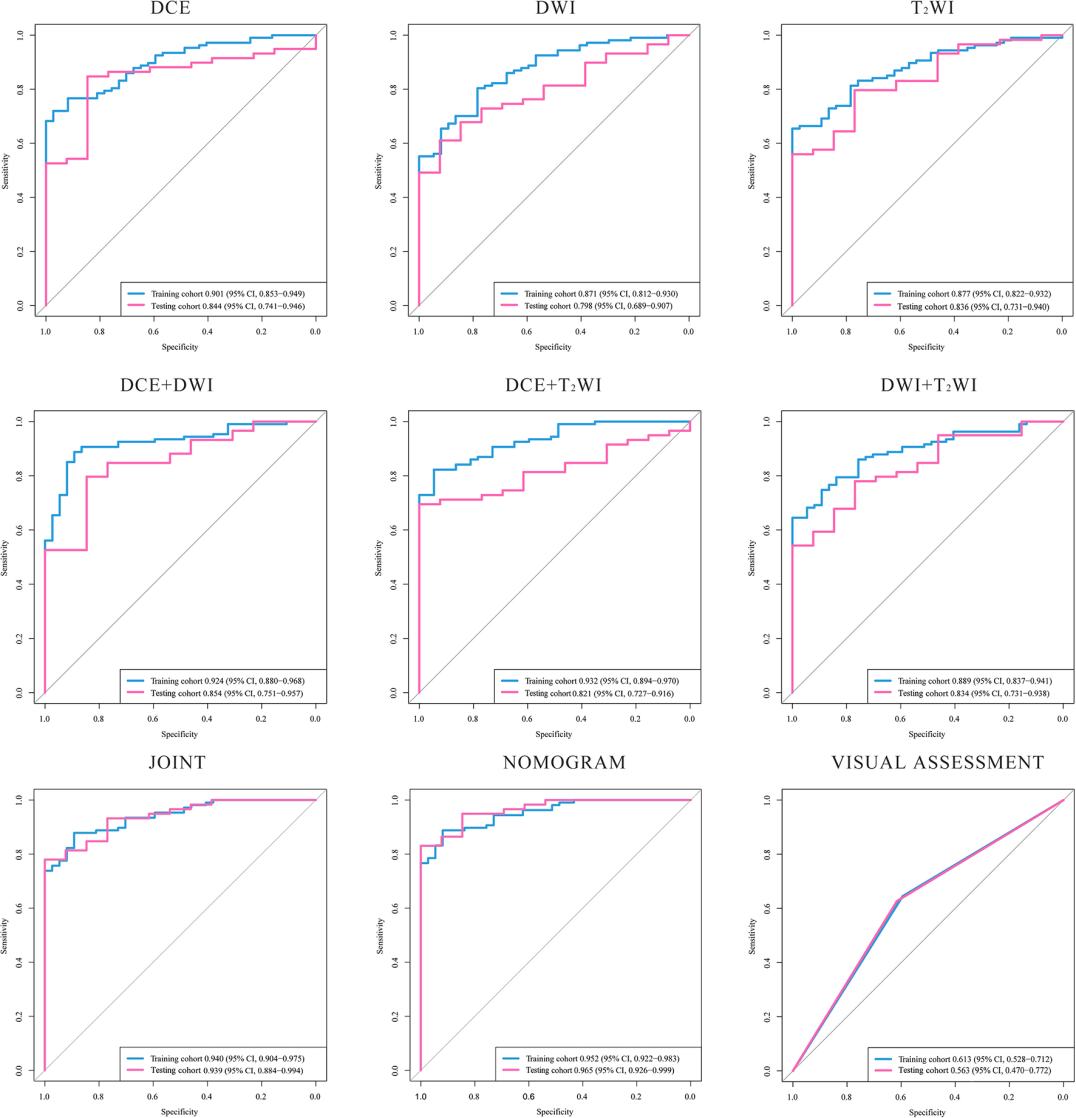
ROC curves of the models. The ROC curves generated by models based on: DCE, DWI, T_2_WI, DCE+DWI. DCE+T_2_WI, DWI+T_2_WI, DCE+DWI+T_2_WI, nomogram combined age and the radiomic signature and the visual assessment of the radiologists. DCE, dynamic contrast enhanced imaging; DWI, diffusion weighted imaging; T_2_WI, _2_ weighted imaging.

By comparing the ROC curves obtained after building radiomics prediction models for single sequences separately and for every two sequences combined with the ROC curve of radiomics prediction models built by combining the three sequences, the radiomics models built by combining the DCE, DWI, and T_2_WI sequences obtained a significant performance improvement. Also, the AUC value of the model based on three sequences combined is the highest among all AUC values we have got. The performance of our joint prediction model is significantly improved, especially in terms of the specificity of the model prediction. The results are shown in **Table 3**. The ROC curves are shown in **Figure 2**.

**Table 3:** Results of the radiomic models and the models based on the nomogram and visual assessment.

	AUC		Accuracy		Sensitivity/Recall		Specificity		Precision		MCC	
	Training	Testing	Training	Testing	Training	Testing	Training	Testing	Training	Testing	Training	Testing
	(95% CI)	(95% CI)										
DCE	0.901	0.844	0.806	0.819	0.804	0.864	0.811	0.615	0.925	0.911	0.561	0.444
	(0.853-0.949)	(0.741-0.946)										
DWI	0.871	0.798	0.847	0.801	0.846	0.814	0.865	0.769	0.947	0.941	0.651	0.493
	(0.812-0.930)	(0.689-0.907)										
T_2_WI	0.877	0.838	0.868	0.777	0.879	0.746	0.838	0.846	0.940	0.957	0.679	0.492
	(0.822-0.932)	(0.731-0.940)										
DCE+DWI	0.932	0.821	0.861	0.708	0.860	0.712	0.865	0.692	0.948	0.913	0.675	0.324
	(0.894-0.970)	(0.727-0.916)										
DCE+T_2_WI	0.924	0.853	0.889	0.820	0.907	0.813	0.838	0.846	0.942	0.96	0.721	0.551
	(0.880-0.968)	(0.751-0.957)										
DWI+T_2_WI	0.889	0.834	0.882	0.777	0.869	0.780	0.919	0.769	0.969	0.939	0.730	0.453
	(0.837-0.941)	(0.731-0.938)										
DCE+DWI+T_2_WI	0.940	0.939	0.924	0.931	0.935	0.932	0.892	0.923	0.961	0.982	0.806	0.791
	(0.904-0.975)	(0.884-0.994)										
Nomogram	0.952	0.965	0.896	0.912	0.887	0.932	0.919	0.846	0.969	0.965	0.756	0.737
	(0.922-0.983)	(0.926-0.999)										
Visual Assessment	0.613	0.563	0.632	0.611	0.644	0.627	0.594	0.538	0.821	0.860	0.21	0.130
	(0.528-0.712)	(0.470-0.772)										

The 9 models contain models based on DCE, DWI, T_2_WI, DCE+DWI, DCE+T_2_WI, DWI+T_2_WI, DCE+DWI+T_2_WI, nomogram and visual assessment.

### Assessment of the Radiomics Nomogram

**Figure 3:**
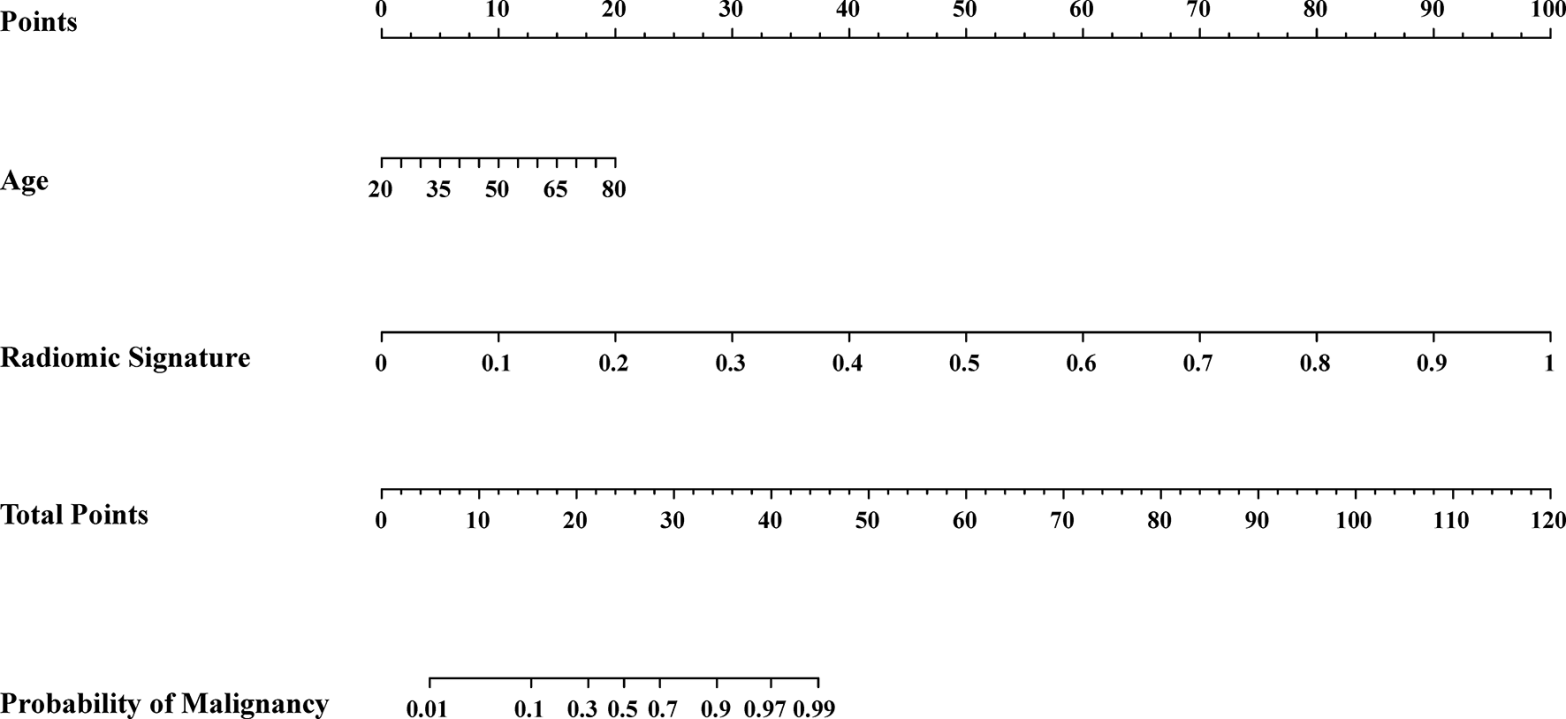
Radiomic nomogram. The radiomic nomogram was conducted based on the patients’ ages from the clinical information and the radiomic signature obtained from the best radiomic model which was based on DCE, DWI and T_2_WI.

**Figure 4:**
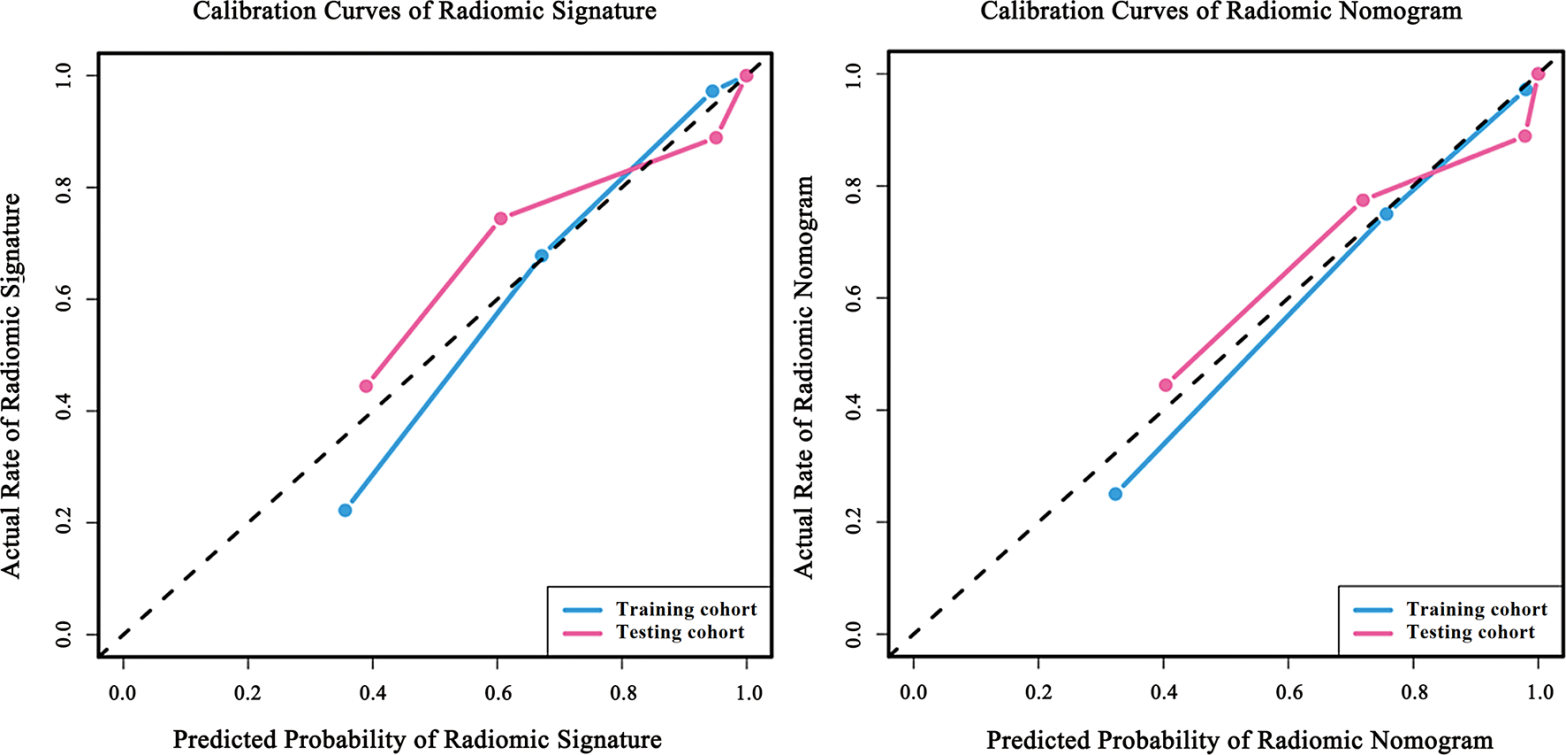
Calibration curves of the radiomic model and the radiomic nomogram. Calibration curves of radiomic signature were built by the radiomic model based on DCE, DWI and T_2_WI. Calibration curves of radiomic nomogram were built by the nomogram. The diagonal line represents the perfect prediction of the ideal model. The blue and pink lines represent the performance of the training and testing cohort in the models, where the models closer to the diagonal line represent better predictions. The calibration curves have gone through the Hosmer-Lemeshow test and have achieved a favorite result.

We conducted the radiomics nomogram by combining the patients’ ages and the radiomics signature. The nomogram has achieved an AUC value of 0.965 (95% CI, 0.926–0.999) and an accuracy of 0.912 in the testing cohort. The radiomics nomogram is shown in **Figure 3**. The calibration curves of the radiomics nomogram and the radiomics model based on DCE, DWI, and T_2_WI are shown in **Figure 4**.

### Comparison of the Prediction Results Between Radiologists and the Radiomics Prediction Model

Two radiologists who have reviewed the in-group patients’ images reached a good consensus of their visual diagnosis upon the DCE, DWI, and T_2_WI sequences’ combination. The results from the radiologists have been used to make a further assessment. According to the model built with the visual assessment achieved from the experts, the AUC value is 0.563 (95% CI, 0.470–0.772), and the accuracy is 0.611 in the testing cohort. The results of the Delong test showed that the predictive AUC values and accuracy of the radiomics prediction model with the combination of the three sequences were significantly higher than those of the prediction model built by the experience of radiologists.

## Discussion

In this study, we conducted a study on the relationship between the malignancy of BI-RADS 4 breast lesions and the imaging features in DCE, DWI, and T2WI MRI, and developed a radiomics prediction model based on MRI. It has been proved that the model has a stronger predictive ability than radiologists’ empirical predictions and can accurately identify the benign and malignant BI-RADS 4 breast lesions, which has application value (22).

Breast MRI has the advantages of good soft-tissue resolution and no radiation and is significantly better than mammography and ultrasonography for early diagnosis and local staging of breast cancer (23, 24). Because MRI is insensitive to microcalcifications and requires a high degree of magnetic field homogeneity, it is easy to cause a false-negative diagnosis (25). Inaccuracies in visual assessment can also lead to overdiagnosis of patients. As a definition from the NCCN guidelines points out, the possibility of the occurrence of BI-RADS 4 breast lesion malignancy range from 2% to 95%, but the actual PPV of breast lesion ranges from 25.7% to 59.2% (26–28). Also, the radiomics model we developed combines multimode MRI images to provide diagnostic doctors and oncologists with a quantitative evaluation tool with greater reliability.

In summary, our study focuses the concentration on developing a way to predict malignant breast lesions through imaging data without causing trauma to the patients.

Among the finally obtained features after extraction and compression by the LASSO logistic regression, the number of T_2_WI image features is the largest, the coefficient value of DCE image features is the largest, and only one DWI feature is selected and the correlation coefficient of this feature is relatively small, which shows that the image features of T_2_WI and DCE have a greater influence on the benignity and malignancy of breast lesions. Compared with other parametric images, T_2_WI MRI images are more likely to reflect the cyst, the margins of the lesion, and the surrounding lymph nodes (29). Therefore, T_2_WI images are often used to detect benign lesions (12). DCE scanning is the most common scan used to diagnose breast disease in clinical practice; it provides higher sensitivity, however, its specificity is variable (30). It can be concluded that T_2_WI scanning and DCE scanning have some complementary properties, and therefore a prediction model combining both sequences can yield substantial performance improvements.

From the features we screened out, wavelet-filtered small area low gray-level emphasis texture feature in the T_2_WI sequence and wavelet-filtered correlation texture feature in the DCE sequence was the most descriptive for breast BI-RADS4-like suspicious lesions. Small area low gray-level emphasis is one of the gray-level size zone matrix (GLSZM) features, which describe the amount of homogeneous connected areas within the volume of a certain size and intensity, thereby describing lesion heterogeneity at a regional scale (26). The grayscale area size matrix is the primary form of the Thibault matrix, which is an advanced statistical matrix of texture features and a powerful tool for medical image analysis. The more homogeneous the image texture is, the larger and flatter the matrix width is. Unlike the stroke and cooccurrence matrices, the GLSZM does not require multiple directional calculations. Specifically, GLSZM is effective in characterizing texture consistency, nonperiodic or speckled textures, and has better performance than granularity, stroke matrix, and cooccurrence matrix for cell nuclei, and dermis (27). Wavelet filtered correlation texture feature is one of the gray level cooccurrence matrix (GLCM) features, which describes the joint distribution of two pixels that have some spatial location relationship (28). The correlation features to measure the degree of similarity of the elements of the spatial grayscale cooccurrence matrix in the row direction. Thus, the local grayscale correlation in the image can be seen from the correlation value magnitude. When the values of matrix elements are uniformly equal, the correlation value is large; contrarily, the correlation value is small when the matrix pixel values differ significantly. If there is a horizontal texture in the image, the correlation value of the horizontal matrix is larger than that of the rest of the matrixes.

Another noteworthy strength of this study is our image segmentation method. The images used in this study were automatically segmented by computer using an optimized deep learning model and then corrected and refined by 2 professional radiologists, so our regions of interest segmentation have a high degree of accuracy and precision. This shows that the image feature values we extracted in this research-based learning are also more convincing. As for image feature values are the basis for the establishment of our prediction model, so this is one of the reasons that can prove that the prediction denseness has application value.

In addition, to demonstrate that this prediction model can reach a higher level than physicians’ diagnosis, we invited two radiologists who have more than 10 years of experience in breast cancer. The two doctors had no prior information about the patient’s personal information and real diagnosis; they were able to determine the benignity or malignancy of the patient’s breast lesions by combining only three sequences of MRI images. The results confirmed that the AUC value, accuracy, sensitivity, specificity precision, and MCC of our developed radiomics prediction model were much higher than those of the radiomics experts.

Medical imaging technology is able to captures a vast amount of information, but most of information was reported in a qualitative and quantitative way. Prospective studies indicated that the computer aided detection (CAD) system constructed using extracted and selected features can effectively distinguish benign and malignant breast lesions (31, 32). Chen et al. (33) extracted a large number of dynamic features from the temporal enhancement pattern of a tumor. Zheng et al. (34) extracted dynamic enhancement and architectural features and spatial variations of pixelwise temporal enhancements from MRI images. Although these studies had also achieved good results, due to the development of medical imaging technique, these features cannot contain rich information on tumors. In 2012, Lambin et al. (17) formally proposed radiomics which attracted the attention of many computer scientists, radiologists, and oncologists. Liu et al. (22) used deep learning to extract features from mammography-based in predicting malignancy. Karen et al. (35) used datasets that contained 64 lesions DCE-MRI images and extracted 38 radiomics features from each image to build SVM models which is able to distinguish the malignant and benign lesions. However, the datasets in these reports are all single modality. Extracting more features can discover the connection between deeper features and the training task. The higher dimensional radiomics features can more fully express tumor heterogeneity, and these features can in part describe the characteristics of breast cancer based on their usefulness, predictive power and uniqueness. In our research, we extracted 3,474 radiomics features from a combination of three sequences (DCE, DWI, T2WI) MRI images to build the model. Thus, we have achieved a satisfactory result.

Our research results confirmed that the multimodal fusion models can complement each other. When one modality cannot obtain obvious information of a single modality, another modality can provide weak supervision information for it. Among the single-sequence models, the model constructed based on DCE scanning technology has the highest AUC. DCE sequence cannot only clearly show morphologic and hemodynamics features of the lesion (36) but also have more significance for the sharpening of the shape and scope of the lesion according to various manifestations such as enhancement mode, blood supply, and cell composition of the lesion. In the dual-sequence combined model, the DCE+T2WI model has higher model evaluation performance than the single-sequence model in terms of accuracy, specificity, and sensitivity. The signal intensity of T2WI is directly related to the shape of the underlying lesion and is usually used to exclude cysts, intramammary lymph nodes, and other benign breast lesions to improve the specificity of diagnosis (37). Among all the models in this study, the best model effect is DCE+T2WI+DWI. Each model of a single sequence has advantages and disadvantages, but the fusion of the three sequences can assist each other (37, 38). By comparing the single-sequence model with the multisequence model, it was found that the multimodal fusion models had better performance in predicting the benign and malignant of BI-RADS 4 breast lesions.

Upon reflection, although this study provides significant benefits, it also has some limitations worth discussing. Firstly, this is a single-center study. Therefore, the lack of multicenter data fusion analysis might affect the generalization ability of the model to a certain extent. Secondly, this prediction model is based on three sequences of images from breast MRI and can combine the characteristics of different images for prediction. However, breast ultrasonography and mammography are also two major means of detecting breast disease (39, 40). Because of the higher cost of MRI, the more restrictive population, and the limitations of an incomplete examination, mammography, and ultrasound scans are even more routinely used as clinical tests (41, 42). So, the absence of these two medical images in this study could potentially cause our model to be less representative, as our prediction model could not be applied to these two sequences of images.

In conclusion, the model based on DCE, DWI, and T_2_WI combined is the most effective in predicting the benignity and malignancy of BI-RADS 4 suspicious lesion.

## Data Availability Statement

The datasets presented in this article are not readily available because “Due to the privacy of patients, the MRI data and clinical information related to patients cannot be available for public access”. Requests to access the datasets should be directed to the corresponding author.

## Ethics Statement

The studies involving human participants were reviewed and approved by the Ethics Committee of Cancer Hospital, Chinese Academy of Medical Sciences. The patients/participants provided their written informed consent to participate in this study.

## Author Contributions

ReZ and WW: study concept, acquisition, analysis and interpretation of data, statistical analysis, and drafting of the article. RL and JL: acquisition, analysis and interpretation of data, statistical analysis, and drafting of the article. ZZ: study concept, acquisition, analysis and interpretation, and critical revision of article. MM and RuZ: acquisition and analysis of data. XZ: study concept and design, interpretation of data, and drafting and critical revision of the article. All authors revised the manuscript critically for important intellectual content and approved the final submitted version.

## Funding

This work was sponsored by the National Natural Science Foundation of China (81901827), Key project of Beijing Hope Marathon Special Fund from China Cancer Foundation (LC2018A20), Natural Science Basic Research Program of Shaanxi (2020JQ-836, 2020JQ-837), Xi’an Polytechnic University (BS201987), and National Key Research and Development Plan of China (2017YFC1309102).

## Conflict of Interest

The authors declare that the research was conducted in the absence of any commercial or financial relationships that could be construed as a potential conflict of interest.

The reviewer KZ declared a shared parent affiliation with several of the authors, ReZ, JL, XZ, to the handling editor at time of review.

## Publisher’s Note

All claims expressed in this article are solely those of the authors and do not necessarily represent those of their affiliated organizations, or those of the publisher, the editors and the reviewers. Any product that may be evaluated in this article, or claim that may be made by its manufacturer, is not guaranteed or endorsed by the publisher.
